# *Clostridium perfringens* Septicemia and a Bleeding Ulcer of a Jejunal Interposition: A Case Report and Short Review of the Literature

**DOI:** 10.1155/2018/4278904

**Published:** 2018-01-22

**Authors:** W. Wild, F. Bormann, H. Sweiti, N. Tamimi, D. Pichulek, M. Divo, P. Dörr, M. Schwarzbach

**Affiliations:** ^1^Department of General, Visceral, Vascular and Thoracic Surgery, Clinical Center Frankfurt Höchst, Frankfurt, Germany; ^2^Department of Trauma and Orthopedic Surgery, BG Trauma Center, Frankfurt, Germany; ^3^Department of Anesthesiology and Emergency Medicine, Clinical Center Frankfurt Höchst, Frankfurt, Germany; ^4^Department of Pathology, Clinical Center Frankfurt Höchst, Frankfurt, Germany; ^5^Institute of Radiology, Clinical Center Frankfurt Höchst, Frankfurt, Germany

## Abstract

**Introduction:**

We report a case of *Clostridium perfringens* septicemia in a patient presenting with a bleeding ulcer of a jejunal interposition.

**Case Presentation:**

An 81-year-old female patient was acutely admitted to our hospital due to hematemesis and melena. She had a history of metastatic gastrointestinal stromal tumor, for which she was receiving second line treatment with sunitinib. She had also undergone a Merendino procedure 4 years prior to presentation. The patient underwent emergency gastroscopy, which revealed a bleeding ulcer in the jejunal interposition. Despite initial endoscopic control of the bleeding and transfusion of blood products, the hemoglobin level continued to drop, and the patient was treated for an assumed hemolytic transfusion reaction. The patient died 3 days following admission, and the results of blood cultures later confirmed a *Clostridium perfringens* septicemia. The postmortem examination revealed a diffuse spread of *Clostridium perfringens* to multiple organs.

**Conclusion:**

This case is a reminder of the importance of considering septicemia, particularly in association with *Clostridium perfringens*, as a potential cause of hemolysis. It also demonstrates the extent of organ involvement in a case of diffuse clostridial myonecrosis.

## 1. Introduction

*Clostridium perfringens* (*C. perfringens*) is an anaerobic, gram-positive bacillus which occurs naturally in the human gastrointestinal and urogenital tract. *C. perfringens* septicemia is a rare but often fatal condition which commonly originates in the uterus, colon, or biliary tract. Most cases report patients who are immunocompromised, with underlying malignant disease, diabetes mellitus, or prior abdominal and retroperitoneal surgery [1–4].

*C. perfringens* is known to release 4 toxins which are highly toxic and primarily responsible for disease pathogenesis. While the beta toxin is known to play a significant role in the pathogenesis of necrotizing enteritis, the alpha toxin is considered responsible for myonecrosis of infected tissues and is also capable of causing hemolysis. In this case, the alpha toxin splits lecithin to phosphocholine and diglyceride, causing impairment of the functional integrity of the red blood cell membrane [5, 6].

Septicemia associated with *C. perfringens* is a lethal condition, with a mortality rate of 70–100% without prompt diagnosis and therapy. The recommended treatment includes aggressive surgical debridement combined with high-dose antibiotics, commonly a combination of penicillin G with clindamycin [6–8]. Some physicians, however, prefer imipenem combined with clindamycin [9, 10].

## 2. Case Report

**Figure 1:**
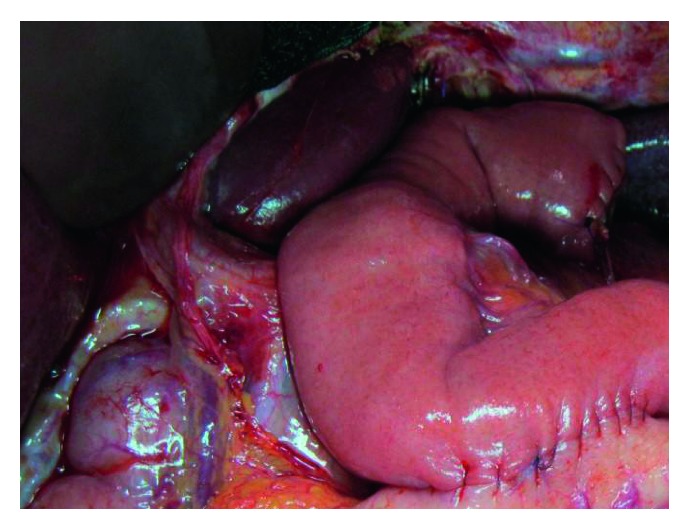
Intraoperative image after resection of the proximal third of the stomach and jejunal interposition.

An 81-year-old female patient presented to our emergency department with acute onset of hematemesis and melena. On admission, the patient appeared to have a poor general health condition and was hemodynamically compromised. Her initial laboratory exams revealed a hemoglobin concentration of 7.7 mg/dl without leukocytosis or C-reactive protein (CRP) elevation. The patient had a history of a 6.5 cm gastrointestinal stromal tumor (GIST) of the cardia, for which she initially received downsizing treatment with imatinib 400 mg/d, followed by surgical resection of the gastroesophageal junction and reconstruction with a jejunal interposition (Merendino procedure) 4 years earlier (Figure 1). Histopathological studies of the tumor revealed a T3-tumor with a positive c-Kit mutation in exon-11 and a Ki-67 proliferation rate of 15%. The risk for disease progression based on Miettinen's criteria was determined as high (size > 5 cm; mitosis rate > 5/HPF), and the patient was subsequently placed on 1st line adjuvant therapy with imatinib 400 mg/d [11]. After two years on 1st line treatment, the patient developed hepatic and peritoneal metastasis and was placed on sunitinib as 2nd line therapy for metastatic GIST. Treatment with a proton pump inhibitor (PPI) was suspended for an unknown reason two years prior to presentation.

**Figure 2:**
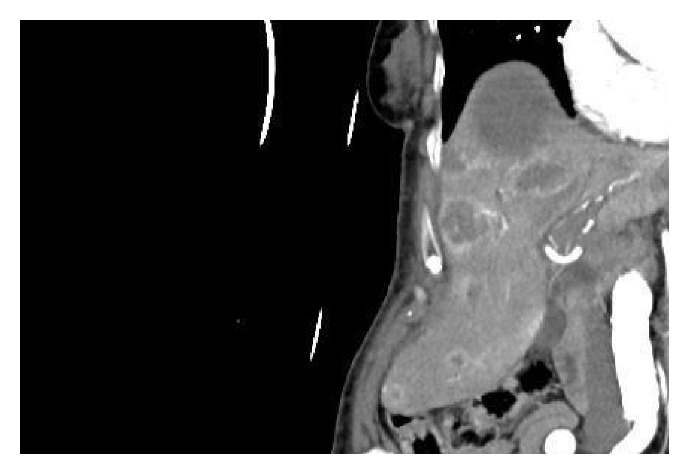
CT scan after endoscopic hemostasis demonstrates liver metastasis and the tumor recurrence in the remnant stomach (frontal image in arterial phase).

The patient was immediately transferred to our intensive care unit, where she was intubated and received two units of packed red blood cells (pRBCs). An emergency gastroscopy was carried out, which revealed active bleeding from a vessel stump in the jejunal interposition, corresponding to a Forrest stage I b upper gastrointestinal bleed. The bleeding was successfully stopped endoscopically by local injection of adrenaline and the application of polymer powder. A CT scan of the thorax and abdomen showed no signs of active bleeding or free abdominal fluid (Figure 2). The known hepatic and peritoneal metastasis were described as constant in size, but increasingly necrotic compared to a previous CT scan. Due to a renewed drop in the hemoglobin concentration during the course of the day, a repeat gastroscopy was performed. This time, it showed diffuse bleeding without a circumscribed source. As a result, we acted to stabilize the coagulopathy by transfusing the patient with 9 units of pRBCs, 6 units of fresh frozen plasma (FFPs), and 6 units of platelet concentrates. In addition, the patient received 3 g of fibrinogen and 4000 IU of PPSB^®^, a prothrombin complex concentrate containing the coagulation factors II, VII, X, and IX.

On the second day after admission, a temporary improvement in the clinical condition of the patient was observed. It was possible to extubate the patient, who was hemodynamically stable with no signs of active bleeding. A phase of atrial fibrillation was cardioverted following treatment with a beta blocker, digoxin, and amiodarone. On the third day following her admission, the patient's condition deteriorated rapidly with the occurrence of fever, gross hematuria, and decreased oxygen saturation. A delayed hemolytic transfusion reaction was suspected, and positive Rh antibodies (anti-c antibody) were detected. Clinically as well as biochemically, the patient was suffering from a hemolysis with a decline of the hemoglobin concentration to 4.8 mg/dl and an increase of lactate dehydrogenase (LDH) to 3842.0 U/l. Given the lack of a septic focus, only a marginal increase in the inflammatory parameters, and pending blood culture results, no antibiotic treatment or surgical therapy was initiated. Due to imminent respiratory failure, the patient was reintubated. Vasopressors, atropine, and crystalloid solutions were administered to treat bradycardia and shock. However, the patient died on the same evening, following an unsuccessful cardiopulmonary resuscitation.

**Figure 3:**
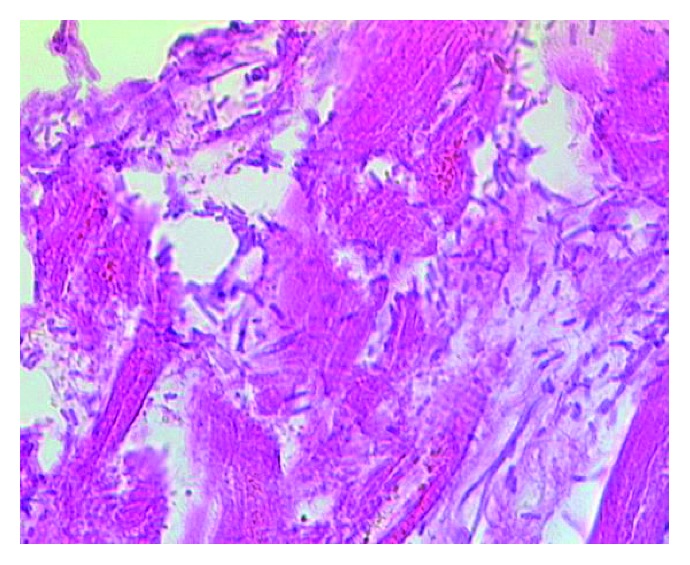
Histological image of bacilli with features consistent with those of *Clostridium perfringens* in the cardial muscle tissue (hematoxylin and eosin, x400).

**Figure 4:**
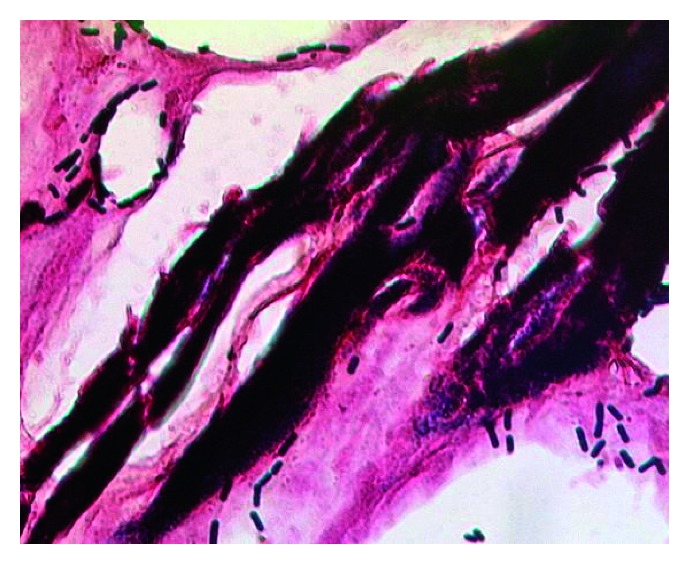
Histological image of *Clostridium perfringens* in the cardial muscle tissue (Gram's stain, x400).

**Figure 5:**
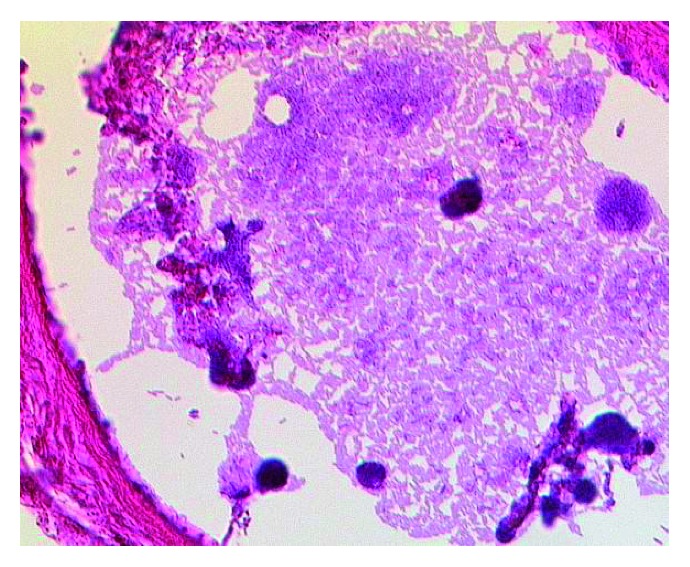
Histological image of bacilli with features consistent with those of *Clostridium perfringens* in lung parenchyma (hematoxylin and eosin, x400).

The results of blood cultures taken on the day of the patient's death revealed gram-labile rods without bacterial growth after two days. The subsequent external analysis confirmed bacteremia with *C. perfringens* and the detection of the alpha toxin gene by polymerase chain reaction, but without any traces of the beta toxin, enterotoxin, epsilon toxin, or iota toxin. The autopsy of the patient revealed a 6 cm sized local recurrence of the GIST and multiple necrotic liver metastases. In addition, a diffuse spread of *C. perfringens* in multiple organs with advanced tissue lysis was histologically confirmed (Figures 3–5). The mucosal ulcer of the jejunal interposition was located 1.5 cm distal to the esophagojejunal anastomosis, which itself was intact. Death due to a septic-toxic shock caused by *C. perfringens* sepsis was determined as the cause of death. A contamination of the administered blood products with *C. perfringens* as the source of the infection was excluded by a subsequent analysis, which was confirmed by an external laboratory.

## 3. Review of the Literature

The majority of clinical data published on *C. perfringens* infections is limited to case reports and retrospective series. Bodey et al. reported results of 136 cases of bacteremia caused by various *Clostridium* species in cancer patients over a period of 12 years. Twenty-seven patients were infected with *C. perfringens*, and only 2 patients suffered from hemolysis. Of these 2 patients, one had colorectal cancer, and the other had acute leukemia with multiple gastric and esophageal ulcera, yet without active bleeding [12]. Both patients died within 24 hours after the onset of the symptoms. In another retrospective analysis of over 200,000 bacteriological records, 33 patients had positive samples for *C. perfringens* (0.017%), and only 1 patient suffered from acute hemolysis [13].

The reason why a natural inhabitant of the human bowel induces such a disastrous infection is still unclear. It appears that, in many cases, the patients are immunocompromised. In their literature review of 40 cases between 1990 and 2010, van Bunderen et al. reported an underlying hematological disorder in 22.5% of cases, a pancreatic or gastric cancer in 12.5%, and/or diabetes mellitus in 30.0% [1]. The most commonly reported origin of infection was hepatobiliary (45.0%), followed by infection after invasive intestinal or gynecological procedures [1]. Juntermanns et al. reported a fatal case of a fulminant septic shock due to a *C. perfringens* skin and soft tissue infection 8 years after liver transplantation [10].

The trigger for the development of a *C. perfringens* infection is as unclear as the method of dissemination in the body. Law and Lee presumed a necrosis of the rectal cancer of their patient as the gate for systemic infection [14]. Hemolysis and septicemia with *C. perfringens* due to emphysematous cholecystitis with subsequent cholestasis, cholangitis, and liver abscesses have also been described [8]. Rajendran et al. suggested a bacterial translocation from the gastrointestinal tract seeding to distant sites like the gallbladder and the liver in his case report [15]. In this present case, we presume the bleeding ulcer in the jejunal interposition to be the potential gate for *C. perfringens* dissemination in an immunocompromised patient. Two gastroscopies on day 1, which involved the endoscopic injection of adrenaline as well as air insufflation to increase the intraluminal pressure, may have contributed to the entry or spread of *C. perfringens* from the gastrointestinal lumen into the tissue. The presence of necrotic GIST metastases may have also facilitated further dissemination in the entire body. Nevertheless, bacterial entry through sources other than an ulcer cannot be excluded with certainty. In their case report, Severin et al. described a patient with *C. perfringens*-associated necrotizing enteritis, where no ulcers were found in postmortem examination [16]. Furthermore, hemorrhagic events of the gastrointestinal tract as well as perforations under targeted therapy with sunitinib have been previously reported [17]. The suspension of PPI therapy 2 years prior to presentation may have also increased the risk for the development of an ulcer. Prophylactic PPI therapy and regular endoscopic monitoring are often recommended following proximal gastric resection and similar procedures [18].

## 4. Conclusion

“This is a disease that begins where other diseases end, with death [19].”

*C. perfringens* septicemia does not leave much time for the diagnosis to be thoroughly considered or ruled out. In our case, the misinterpretation of hemolysis as a transfusion reaction resulted in other differential diagnosis being disregarded. The lack of septic focus and the marginal increase in inflammatory parameters further supported this misinterpretation with the consequence of a missed antibiotic therapy. *C. perfringens* septicemia should always be considered as a potential cause for hemolysis.
